# Activation of Myeloid Dendritic Cells by Up-Regulating RAGE/JAK/STAT Pathway Induced by Cigarette Smoke Exposure in Mice With Emphysema

**DOI:** 10.1155/mi/5595989

**Published:** 2025-09-04

**Authors:** Jinglin Gao, Huijuan Wang, Guang Zhou, Zhou Zhou, Caixia Liang, Xiaoning Zhong

**Affiliations:** ^1^Department of Respiratory and Critical Care Medicine, The First Affiliated Hospital of Guangxi Medical University, Nanning 530021, China; ^2^Liuzhou Key Laboratory of Prevention and Treatment of Rheumatic Diseases, The Fourth Affiliated Hospital of Guangxi Medical University, Liuzhou 545005, China; ^3^Medical Care Ward, Guilin People's Hospital, The Fifth Affiliated Hospital of Guilin Medical University, Guilin 541002, China

**Keywords:** cigarette smoke exposure, conditional knockout, emphysema, myeloid dendritic cells, RAGE

## Abstract

**Objective:** To explore the potential role of the RAGE/JAK/STAT pathway along with the activation of myeloid dendritic cells (mDCs) and B cells induced by cigarette smoke exposure in mice.

**Methods:** 57BL/6J mice and RAGEfl/flCD11c-Cre mice were subjected to cigarette smoke for 24 weeks and mated with room air controls. Mice bone marrow-derived dendritic cells (BMDCs) were treated with cigarette smoke extracts (CSEs), CSE with the RAGE inhibitor FPS-ZM1 or CSE with the JAK2 inhibitor AG490. The extent of emphysema in these mice was assessed using the average alveolar lining distance (Lm). Real-time PCR was employed to quantify the mRNA expression levels of RAGE, JAK2, STAT1, STAT3 and STAT5 in lung tissue samples. The levels of IL-6 and IL-1β in mouse serum and BMDC supernatant were quantified using ELISA. Flow cytometry was employed to measure the expression of CD40, CD86, RAGE, p-JAK2, p-STAT1, p-STAT3 and p-STAT5 of lung mDCs and BMDCs in mice. Flow cytometry was employed to identify markers CD69, CD86 and CD138 on pulmonary B cells.

**Results:** Exposing mice to cigarette smoke triggered an exaggerated pulmonary mDCs response and elevated the RAGE/JAK/STAT pathway in both pulmonary mDCs and lung tissue, correlating with enhanced B cells response in lungs. Conditional knockdown of RAGE on dendritic cells (DCs) resulted in a reduction of activity within JAK/STAT pathway, impeded the exaggerated mDCs and B cells responses induced by smoking, down-regulated the serum inflammatory response and mitigated emphysema in cigarette smoke-exposed mice. Within a regulated laboratory setting, BMDCs were activated, leading to the amplification of the RAGE/JAK/STAT pathway in these cells after CSE exposure. FPS-ZM1 and AG490 reduced inflammatory factors in the supernatant and activation of BMDC.

**Conclusion:** In mice, prolonged exposure to cigarette smoke triggers the activation of mDCs by enhancing the RAGE/JAK/STAT pathway. Conditional knockdown of RAGE on DCs can prevent the activation of mDCs and B cells triggered by cigarette smoke, indicating that RAGE could be a potential target for treating smoking-induced emphysema.

## 1. Introduction

Chronic obstructive pulmonary disease (COPD) is a heterogeneous pulmonary condition characterised by a progressive obstruction of airflow due to structural abnormalities in the alveoli and airways [1]. Globally, COPD affects approximately 400 million individuals and is the third leading cause of mortality [2]. Tobacco use is a major environmental contributor to COPD [3]. COPD is a preventable and treatable incompletely reversible chronic disease mainly involved in remodelling and airways narrowing, alveolar and pulmonary vascular damage leading to emphysema [4]. In smokers, damage may be confined to either major or minor airways; however, some individuals continue to experience persistent alveolar damage resulting in emphysema. In these individuals, chronic inflammation of the pulmonary airways and alveolar injury are associated with adaptive immune responses [5–7]. This pathological process involves a variety of inflammatory cells, including macrophages, dendritic cells (DCs), neutrophils, eosinophils and various lymphocyte subtypes and more than 100 different inflammatory mediators [8–12]. Recent research indicates that DCs are crucial in linking processes involved in the progression of COPD [13]. That is, the inflammatory response in COPD extends beyond intrinsic immunity, as DCs play a pivotal role in antigen recognition and presentation. This process subsequently activates B and T lymphocytes, thereby initiating the acquired immune response and contributing to the amplification and progression of lung inflammation [14]. However, the specific regulatory mechanisms are not yet clear.

Chronic lung inflammation is exacerbated and persistently advanced in COPD cases caused by smoking when the adaptive immune response is disrupted and onset occurs [7]. Among these, DCs serve as crucial intermediaries. They initiate and coordinate innate and adaptive immunity responses to tissue injury or infection in the lungs [15]. A study revealed that co-culturing myeloid dendritic cells (mDCs) from the lungs of COPD patients with CD4^+^ T cells from peripheral blood resulted in an increased differentiation of CD4^+^ T cells into Th1 and Th17 cells [16]. Conversely, the inhibition of RAGE prevented the differentiation of CD4^+^ T cells into Th1 cells [17], while pre-stimulation with advanced glycation end-products (AGEs) promoted CD4^+^ T cell differentiation [18]. In individuals suffering from COPD, the lungs exhibit a significant presence of B cell lymphoid follicles, accompanied by an observed increase in B cell-derived products (autoantibodies) in both the bloodstream and pulmonary tissue [19]. When comparing non-smokers and smokers without COPD, it has been determined that BAFF expression increased in B cells in COPD patients. BAFF inhibited cigarette smoke extract (CSE)-induced apoptosis by preventing the suppression of nuclear factor-κB (NF-κB) activation caused by CSE. This indicates that BAFF expression in B cells promotes lung B cell activation and LF expansion, thereby establishing a self-perpetuating cycle that exacerbates COPD progression [20]. Additionally, another study showed increased expression of CD69, CD86 and CD138 on B cells following co-culture with antigen-presenting DCs and B lymphocytes. This suggests that DCs enhance B cell activation and differentiation into plasmacytoid cells, a process dependent on the nuclear accumulation of NF-kB/cRel [21]. These results imply that DCs play a crucial role in bridging the innate and adaptive immune systems, and their involvement in initiating B-cell responses may be pivotal in the pathogenesis of COPD.

RAGE is an immunoglobulin superfamily member and serves as an AGE receptor. It interacts with and modulates cellular responses to various damage-associated molecular pattern molecules (DAMPs) like HMGB1 and is crucially involved in numerous chronic inflammatory diseases [22, 23]. Under normal physiological conditions, RAGE is present in minimal amounts under healthy conditions, but its expression markedly increases during prolonged inflammation because of the buildup of different RAGE ligands [24]. When ligands interact with RAGE, they have the potential to trigger multiple signalling cascades, including JAK/STAT, resulting in the stimulation of transcription regulators like NF-κB, STAT3 and others to contribute [25, 26], via an internal positive feedback, converting a short-term inflammatory reaction into prolonged cellular impairment [27]. RAGE contributes to controlling apoptosis, cell growth, differentiation and adhesion within lung tissue. Elevated levels of RAGE in adult lungs are linked to a higher alveolar destructive index and ongoing inflammation, which may culminate in the development of emphysema characterised by tissue destruction [28]. Exposure to tobacco smoke has been shown to increase RAGE levels in both cellular models and mouse lungs, with RAGE facilitating cytokine production through the pro-inflammatory NF-κB pathway. Furthermore, transgenic mice with elevated RAGE expression demonstrated significant alveolar inflammation, resulting in COPD-like changes [29]. RAGE promotes inflammatory responses in the lungs along with massive cytokine activation in vivo. The JAK/STAT pathway, a common reactive mechanism mediated by numerous cytokines, can further amplify and exacerbate pre-existing inflammatory responses through a cascading effect [30]. However, the specific role of RAGE in lung inflammation, particularly its potential involvement in the activation of lung DCs and the initiation of acquired immunity in the context of chronic herb smoke exposure, remains inadequately understood.

In this study, we sought to evaluate the possible involvement of the RAGE/JAK/STAT pathway, along with the heightened responses of mDCs and B cells, induced by cigarette smoke in mice. Additionally, we examined the effects of CSE and inhibitors of RAGE and JAK2 on bone marrow-derived dendritic cells (BMDCs) in vitro.

## 2. Materials and Methods

### 2.1. Animals and Cigarette Smoke Exposure

Mice of the C57BL/6J strain, aged 6–8 weeks, obtained from the Laboratory Animal Centre at Guangxi Medical University, were randomly divided into two groups: the wild type air group (WT-AIR) and the wild type cigarette smoke exposure group (WT-CS). RAGE^fl/fl^CD11c-Cre mice (C57BL/6J, aged 6–8 weeks) produced by Shanghai Southern Model Biotechnology Development Company, Ltd. (Shanghai, China) were randomly assigned to either the RAGE conditional knockout air group (RAGE^fl/fl^CD11c-Cre-AIR) or the RAGE conditional knockout cigarette smoke exposure group (RAGE^fl/fl^CD11c-Cre-CS). Genotypes were determined through PCR amplification of genomic DNA extracted from mouse tail samples. Each mouse was housed in a sanitised cage, provided with sterilised food and granted unrestricted access to water. All live animal experiments were conducted with the approval of the Guangxi Medical University's Animal Ethics Committee. The mice in the CS group were kept in an enclosed 0.75 m^3^ chamber and exposed to Nanning Zhen Long unfiltered cigarettes according to our previously described method [31]. The exposure to cigarette smoke was maintained consistently over a period of 24 weeks. Control mice were housed in an identical chamber, where they were exposed to clean air for the same duration of 24 weeks.

### 2.2. Mice Serum

Following anaesthesia, remove the mice's eyeballs to take blood and leave it in 1.5 mL dry EP tubes. The amount of blood collected from each mouse was about 1 mL, and centrifuged at 3000 rpm for 20 min after standing at room temperature for 2 h, then place the supernatant in the refrigerator at −80°C for ELISA.

### 2.3. Lung Tissue

After fixation, the left lung tissue of mice was stained with haematoxylin and eosin (H&E) for morphological examination. For histological examination, 10 randomly selected areas at 200x magnification were photographed from four distinct regions of each mouse's lung. The average linear intercept (Lm), which represents the typical spacing of alveolar walls and correlates with the extent of emphysema, was measured using light microscopy as outlined in prior studies [32]. Two researchers independently evaluated Lm without access to specific details.

### 2.4. Preparation of CSE

CSE was generated in accordance with the methodologies delineated in our previous research and supplementary studies [33]. Briefly, the emissions from five unfiltered Nanning Zhen Long cigarettes (each containing 12 mg of tar and 0.9 mg of nicotine) were filtered through 5 mL of RPMI 1640 medium. The concentration of CSE was determined using a dual-wavelength violet spectrophotometer (Lambda Bio 20, Perkin Elmer) by measuring absorbance at 320 nm. The CSE mixture was filtered using a 0.22 μm membrane. Cells were exposed to a 0.5% CS extract solution for 3 h.

### 2.5. The Generation and Treatment of BMDC

Bone marrow cells from C57BL/6J mice aged 4–10 weeks were cultured in RPMI 1640 medium supplemented with 10% fetal bovine serum (FBS), 40 ng/mL granulocyte–macrophage colony-stimulating factor (GM-CSF, Peprotech, USA) and 10 ng/mL interleukin-4 (IL-4, Peprotech, USA). The culture medium was partially refreshed on days 2, 4 and 6. BMDC were allocated into four groups: control, CSE, CSE + FPS-ZM1 and CSE + AG490. On the sixth day, the CSE + FPS-ZM1 group was subjected to a 2 h treatment with 5 µM FPS-ZM1 in darkness prior to the administration of 0.1% CSE. Concurrently, the CSE + AG490 group received a 2 h treatment with 10 µM AG490, whereas the CSE group was incubated solely with 0.1% CSE for an identical duration. The control group was maintained in a 5% CO_2_ incubator at 37°C overnight. The medium was also incubated under the same conditions. On the seventh day, the cell culture supernatant was collected, centrifuged and stored at −80°C in preparation for ELISA, while cells were harvested and processed for flow-through assay within 1 h.

### 2.6. Preparation of Single-Cell Suspensions

Individual cells from mouse lungs were isolated using mechanical disruption, enzymatic digestion and centrifugation, as previously described. After removal of blood and intravascular cells, the lung tissue was minced and digested in RPMI 1640 medium containing collagenase type IV (1 mg/mL; Sigma, USA) at 37°C for 40 min. Subsequently, the remaining undigested tissue was homogenised, filtered through a 70 µm filter, centrifuged and red blood cells were removed using RBC Lysis Buffer (Solarbio, China).

### 2.7. Real-Time Quantitative PCR

In accordance with the manufacturer's instructions, mRNA from lung tissue was extracted using Trizol (Invitrogen) to measure the relative transcript levels of RAGE, JAK2, STAT1, STAT3 and STAT5a. cDNA was synthesised using oligo (dT) primers obtained from Novizan Biotechnology in China. Real-time qPCR was conducted using reagents also sourced from Novizan Biotechnology, China, and performed on the Applied Biosystems 7500 platform (ThermoFisher SCIENTIFIC) according to the manufacturer's protocol. The following primers were employed:

5-GGTTGTCTCCTGCGACTTCA-3 and 5-TGGTCCAGGGTTTCTTACTCC-3 for GAPDH; 5-ACTGAAGCTTGGAAGGTCCTC-3 and 5-GATGGGTTCCTCCTTGGGTG -3 for RAGE;

5-GTGTGGAGATGTGCCGCTATGAC-3 and 5-AGTCTCGGAGGTGCTCTTCAGTG-3 for JAK2; 5-GCTGCCGAGAACATACCAGAGAATC-3 and 5-CCAGTTCGCTTAGGGTCGTCAAG-3 for STAT1; 5-AATCTCAACTTCAGACCCGCCAAC-3 and 5-GCTCCACGATCCTCTCCTCCAG-3 for STAT3; 5-AGTCGGTGACGGAGGAGAAGTTC-3 and 5- CGGTGGCAGTAGCATTGTGGTC-3 for STAT5a.

The 2^−ΔΔCt^ technique was utilised to assess mRNA expression levels.

### 2.8. ELISA

Commercial kits from Cloud-Clone (Wuhan, China) were utilised to quantify serum IL-6 and IL-1β levels in mice and BMDC supernatant. After loading standards and samples, 40 μL of sample diluent was added, followed by the introduction of an antibody conjugated with horseradish peroxidase (HRP). The wells were sealed and incubated at 37°C for 60 min, then the liquid was removed and the wells were washed five times. Substrates were added to the wells and incubated at 37°C for 15 min in the absence of light. Optical density was measured using a Thermo microplate reader within 15 min after the addition of 50 μL of stop solution.

### 2.9. Flow Cytometry

Living cells were selected using a stain capable of distinguishing between viable and non-viable cells (fixable viability dye eFluor780, eBioscience). Flow cytometry was employed to assess surface marker expression on both mouse and cell samples, utilising surface staining with anti-mouse-specific antibodies. The antibodies used in this study included PerCP-anti-mouse/human CD45R/B220, PE/Cy7-anti-mouse CD69, PE-anti-mouse CD138, PE-anti-mouse CD40, APC-anti-mouse CD86, PE-anti-mouse MHCII, APC-anti-mouse CD11c (BD Pharmingen), PE-anti-mouse p-Stat1 (Tyr701), PE-anti-mouse p-Stat3 (Tyr705), PE-anti-mouse p-Stat5 (Tyr694) (cell signalling technology), Alexa Fluor 488-anti-mouse RAGE (R&D systems), PE-anti-mouse p-JAK2 (Tyr1007 + Tyr1008), PerCP-eFluor710-anti-mouse MHC-II and FITC-anti-mouse CD11c (eBioscience). For intracellular staining of p-JAK2, p-STAT1, p-STAT3, and p-STAT5, cells were fixed using a pre-warmed fix buffer and incubated for 10 min at 37°C, followed by permeabilization with a perm buffer and incubation for 30 min on ice. The analysis was performed using a BD FACS Verse flow cytometer, and data interpretation was conducted with FCS Express software.

### 2.10. Statistical Analysis

The data were presented as mean ± standard deviation. A one-way ANOVA or an unpaired Student's *t*-test was employed to assess differences between groups. SPSS Statistics version 25.0 and GraphPad Prism 8.0 were used for analysing our data, and a *p*-value of less than 0.05 was considered statistically significant.

## 3. Results

### 3.1. Chronic Emphysema in Mice After Cigarette Smoke Exposure

**Figure 1:**
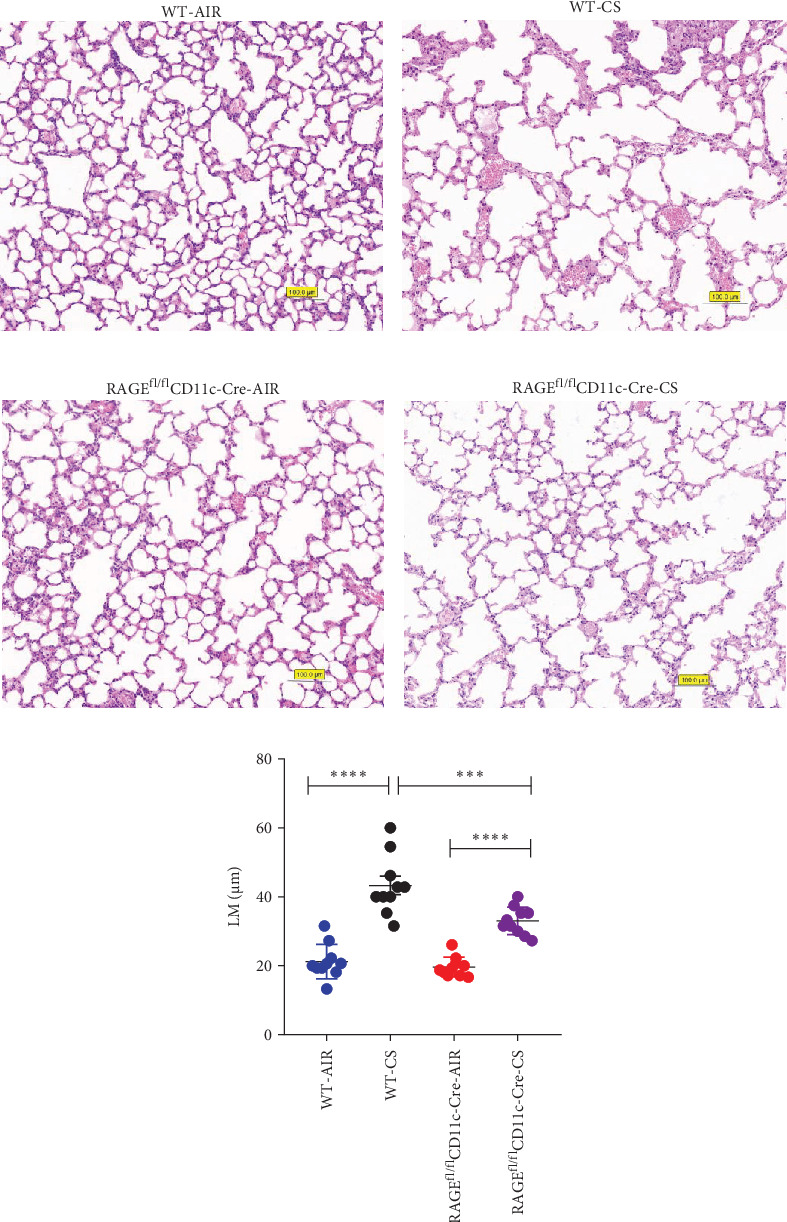
HE images of lung tissue from WT-AIR (A), WT-CS (B), RAGE^fl/fl^CD11c-Cre-AIR (C) and RAGE^fl/fl^CD11c-Cre- CS (D) viewed at 200x magnification. (E) The analysis of Lm of lung tissue in different groups; *⁣*^*∗∗∗*^*p＜*0.001, *⁣*^*∗∗∗∗*^*p* < 0.0001.

After tobacco exposure for 24 weeks, HE staining was performed on histopathological sections of mice lungs tissue and Lm was calculated to assess the severity of emphysema. As illustrated in Figure 1A,B,C, mice subjected to chronic cigarette smoke exposure exhibited significant morphological changes compared to the air-exposed group. These changes included thinning and fracturing of alveolar walls, fusion of alveoli, widening of alveolar spaces, formation of emphysema and a statistically significant increase in Lm (*p*  < 0.05).

Subsequently, RAGE was knocked out in DCs. Compared to the WT-CS group, the RAGE^fl/fl^CD11c-Cre-CS group demonstrated a reduction in Lm and less pronounced emphysema (*p*  < 0.05). However, the Lm value in the RAGE^fl/fl^CD11c-Cre-CS group remained significantly higher than that in the RAGE^fl/fl^CD11c-Cre-AIR group (*p*  < 0.05). These findings suggest a protective effect of knockdown of RAGE in DCs against cigarette-associated emphysema in mice.

### 3.2. Up-Regulation of RAGE/JAK/STAT Pathway in Lung Tissue and Pulmonary mDCs in a Smoking Mouse Model of Emphysema

**Figure 2:**
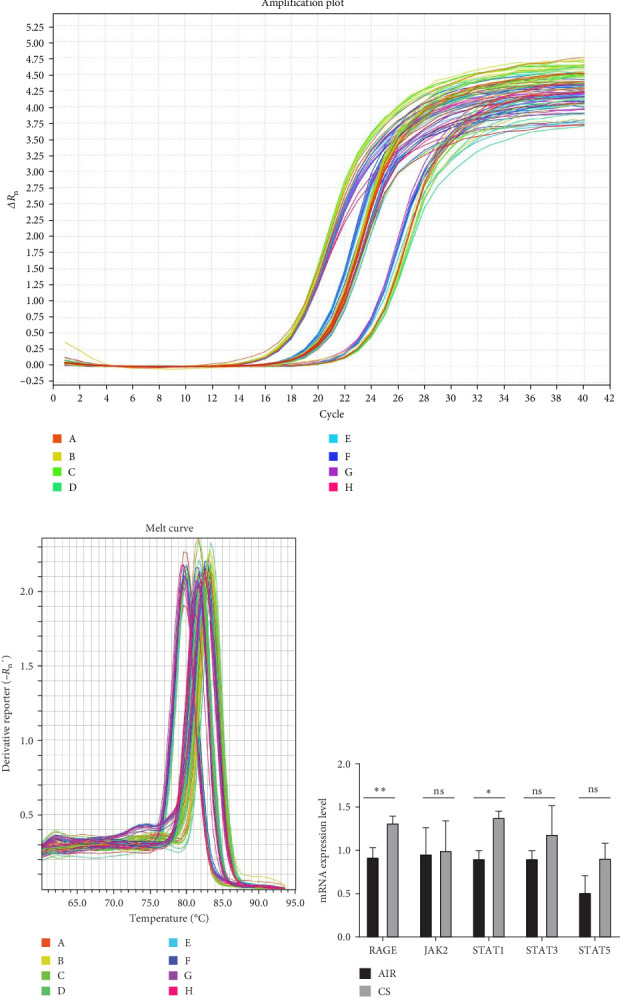
The impact of extended tobacco exposure on the mRNA levels of RAGE/JAK/STAT in murine lung tissue. RT-PCR was used to quantify the mRNA expression levels of RAGE, JAK2, STAT1, STAT3 and STAT5. (A) Amplification plot. (B) Melt curve. (C) The expression levels of mRNA; *⁣*^*∗*^*p* < 0.05, *⁣*^*∗∗*^*p* < 0.01; NS, no significance.

**Figure 3:**
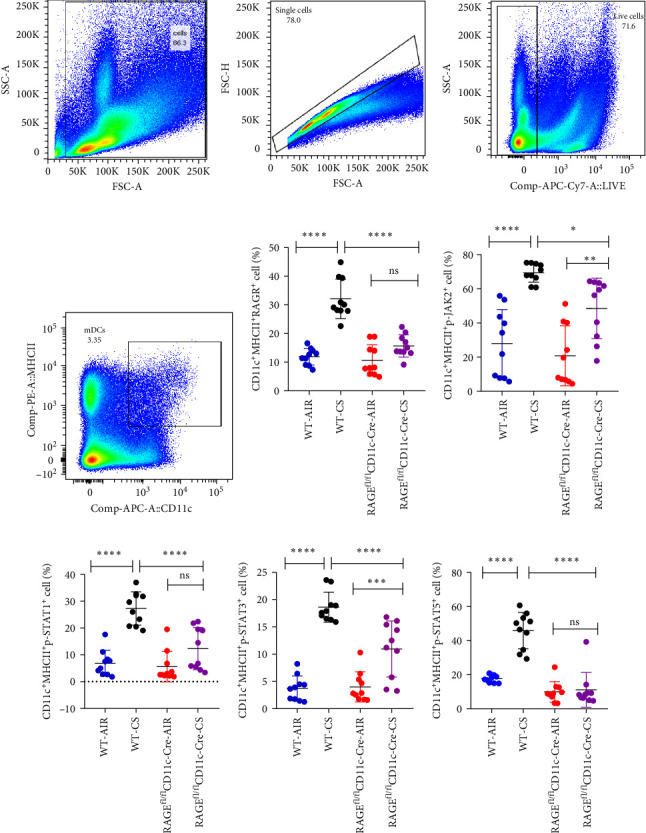
The expression of the RAGE/JAK/STAT in pulmonary mDCs of mice using flow cytometry. Selective deletion of RAGE in dendritic cells can reduce the JAK/STAT pathway. (A) and (B) illustrated the methodology for identifying dendritic cells via flow cytometry. Expression levels of RAGE (C), p-JAK2 (D), p-STAT1 (E), p-STAT3 (F) and p-STAT5 (G) in lung mDCs of both wild type and RAGE^fl/fl^CD11c-Cre mice subjected to AIR and CS were analysed; *⁣*^*∗*^*p* < 0.05, *⁣*^*∗∗*^*p* < 0.01, *⁣*^*∗∗∗*^*p* < 0.001, *⁣*^*∗∗∗∗*^*p* < 0.0001; NS, no significance.

Real-time quantitative PCR analysis revealed a significant increase in the mRNA levels of RAGE and STAT1 in the lung tissue of mice subjected to prolonged tobacco exposure, while the mRNA levels of JAK2, STAT3, and STAT5 remained unchanged (Figure 2). This suggests that cigarette smoke activates the expression of RAGE and STAT1 genes. For further investigation, we performed flow cytometry on prepared single-cell suspensions of lung tissue. FSC-A and SSC-A circled the cells to be analysed, FSC-A and FSC-H excluded adherent cells and fixable viability dye removed dead cells. mDCs were identified through CD11c and MHC II staining (CD11c^+^MHC Ⅱ^+^). We found a significant elevation in the levels of RAGE, phosphorylated JAK2, STAT1, STAT3 and STAT5 in lung mDCs following extended exposure to cigarette smoke (Figure 3). This implies that cigarette smoke exposure enhances the expression of the RAGE/JAK/STAT pathway in pulmonary mDCs in mice. Upon the removal of RAGE from DCs in mice, flow cytometry analysis revealed a significant decrease in the expression levels of RAGE, p-JAK2, p-STAT1, p-STAT3 and p-STAT5 in mDCs within the lungs of the RAGE^fl/fl^CD11c-Cre-CS group compared to the WT-CS group (*p*  < 0.05). The expressions of RAGE, p-STAT1 and p-STAT5 in mDCs from the RAGE^fl/fl^CD11c-Cre-CS group did not show a statistically significant increase compared to the RAGE^fl/fl^CD11c-Cre-AIR group (*p*  > 0.05). Conversely, mDCs in the RAGE^fl/fl^CD11c-Cre-CS group demonstrated significantly elevated levels of p-JAK2 and p-STAT3 relative to the RAGE^fl/fl^CD11c-Cre-AIR group (*p*  < 0.05). These findings suggest that the reduction of RAGE expression in DCs leads to the down-regulation of the JAK/STAT pathway in pulmonary mDCs of mice exposed to cigarette smoke.

### 3.3. Stimulation of the Lung mDCs in Mice With Emphysema due to Cigarette Smoke Exposure

**Figure 4:**
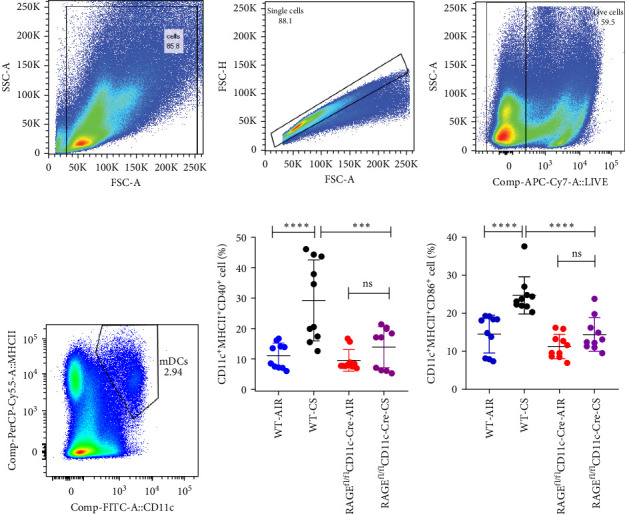
Flow cytometry was employed to assess the activation of pulmonary mDCs in mice. (A) and (B) illustrated the delineation of dendritic cells. The expression of CD40 (C) and CD86 (D) on lung mDCs in wild type and RAGE^fl/fl^CD11c-Cre mice subjected to AIR and CS; *⁣*^*∗∗∗*^*p* < 0.001, *⁣*^*∗∗∗∗*^*p* < 0.0001; NS, no significance.

Flow cytometry analysis revealed that prolonged exposure to tobacco smoke activates pulmonary mDCs in mice. In the chronic cigarette smoke group, the expression of CD40 and CD86 on pulmonary mDCs was markedly elevated compared to the WT-AIR group (*p*  < 0.05, Figure 4), indicating that chronic cigarette smoke exposure enhances the development and activation of pulmonary mDCs. Furthermore, the expression of CD40 and CD80 on pulmonary mDCs in the RAGE^fl/fl^CD11c-Cre-CS group was significantly lower than the WT-CS group (*p*  < 0.05), suggesting that selective reduction of RAGE in DCs results in decreased activity of lung mDCs following prolonged cigarette smoke exposure.

### 3.4. The Improved B Cell Responses in the Lungs of Mice Subjected to Cigarette Smoke

**Figure 5:**
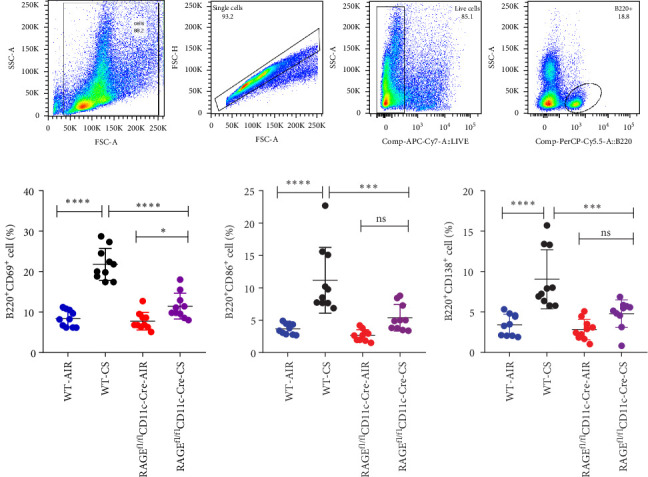
The activation of pulmonary B cells in mice determined by flow cytometry. Conditional knockout of RAGE in DCs can inhibit the activation of B cells. (A) illustrated the delineation of B cells detected by flow cytometry. The expression levels of CD69 (B), CD86 (C) and CD138 (D) in lung B cells from different groups; *⁣*^*∗*^*p* < 0.05, *⁣*^*∗∗∗*^*p* < 0.001, *⁣*^*∗∗∗∗*^*p* < 0.0001; NS, no significance.

Based on flow cytometry analysis, it was observed that the expression levels of CD69, CD86 and CD138 on B cells in murine lung tissue increased following prolonged tobacco smoke exposure, compared to the control group exposed to ambient air (*p*  < 0.05, Figure 5), indicating that prolonged exposure to tobacco smoke enhances the activation and differentiation of B cells into plasmablasts in lungs. Furthermore, the expression levels of CD69, CD86 and CD138 in lung B cells of the RAGE^fl/fl^CD11c-Cre-CS group were higher than those in the RAGE^fl/fl^CD11c-Cre-AIR group (*p*  < 0.05). However, these levels were lower in the RAGE^fl/fl^CD11c-Cre-CS group compared to the WT-CS group (*p*  < 0.05). This study demonstrates that the targeted reduction of RAGE in DCs results in diminished B cell activation in the lungs of mice exposed to tobacco smoke.

### 3.5. Increased Serum IL-6 and IL-1β Levels in Mice After Extended Cigarette Smoke Exposure

**Figure 6:**
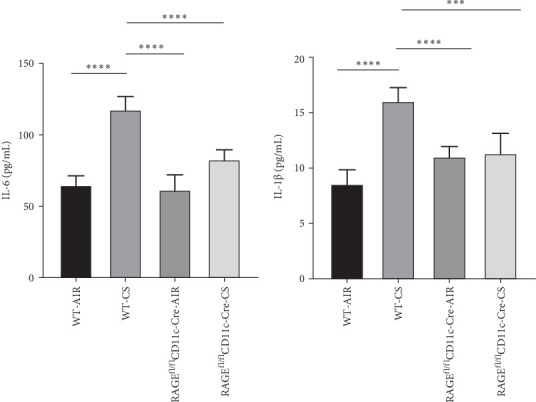
The levels of IL-6 and IL-1β in mouse serum measured by ELISA. Analysis of IL-6 (A) and IL-1β (B) levels in four groups; *⁣*^*∗∗∗*^*p* < 0.001, *⁣*^*∗∗∗∗*^*p* < 0.0001.

ELISA was employed to measure serum inflammatory markers IL-6 and IL-1β in prolonged cigarette-smoking mice. Our findings indicate that prolonged exposure to cigarette smoke elevates serum IL-6 and IL-1β levels in mice, while the selective reduction of RAGE in DCs markedly decreases the secretion of these cytokines (*p*  < 0.05, Figure 6).

### 3.6. The Activation of BMDC Induced by CSE and the Effects of FPS-ZM1 and AG490 Intervention In Vitro

**Figure 7:**
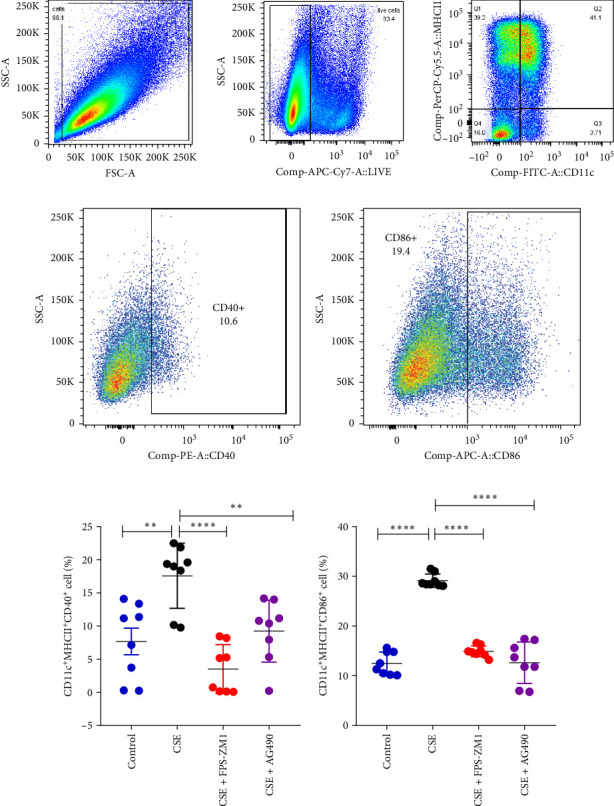
The expression levels of CD40 and CD86 on BMDC measured by flow cytometry. (A) Illustrated how to delineate dendritic cells. The analysis of the expression levels of CD40 (B and D) and CD86 (C and E) markers on BMDC in different groups; *⁣*^*∗∗*^*p* < 0.01, *⁣*^*∗∗∗∗*^*p* < 0.0001.

To evaluate the effects of CSE and the RAGE/JAK/STAT signalling pathway on DCs, BMDC were generated from mice pre-treated with CSE and inhibitors of RAGE (FPS-ZM1) and JAK2 (AG490). Flow cytometry analysis conducted 24 h post-CSE exposure revealed a significant increase in the expression levels of CD40 and CD86 in BMDC compared to the control group. Compared to the CSE group, the levels of CD40 and CD86 in BMDC were notably reduced in the CSE + FPS − ZM1 and CSE + AG490 groups (*p*  < 0.05, Figure 7). These findings indicate that the activation of BMDC was induced by CSE, while the interventions with FPS-ZM1 and AG490 mitigate this CSE-induced activation in vitro.

### 3.7. The Regulation of RAGE/JAK/STAT Pathway in BMDC in Vitro

**Figure 8:**
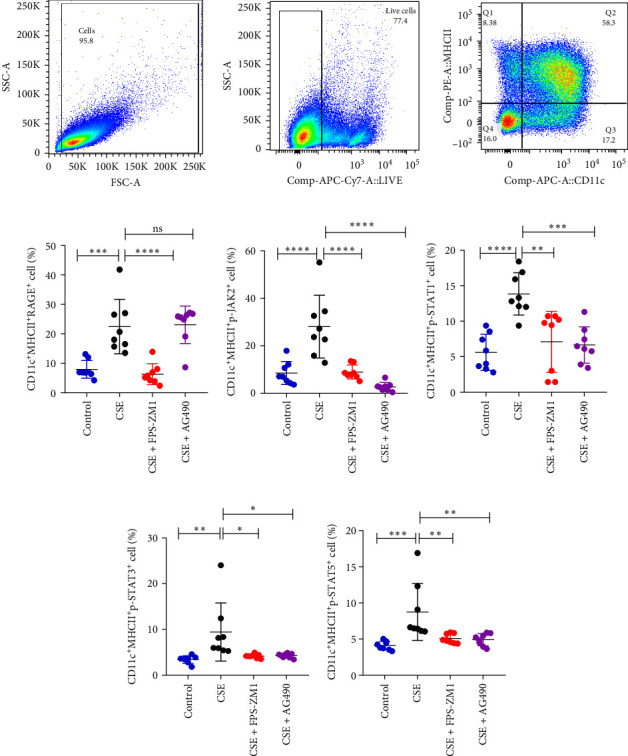
The expression levels of the RAGE/JAK/STAT pathway in BMDC determined by flow cytometry. (A) Illustrated how to delineate dendritic cells. The levels of RAGE (B), p-JAK2 (C), p-STAT1 (D), p-STAT3 (E) and p-STAT5 (F) of BMDC in four groups; *⁣*^*∗*^*p* < 0.05, *⁣*^*∗∗*^*p* < 0.01, *⁣*^*∗∗∗*^*p* < 0.001, *⁣*^*∗∗∗∗*^*p* < 0.0001; NS, no significance.

The BMDC were prepared following previously established protocols. Relative to the control group, there was a significant upregulation of RAGE, p-JAK2, p-STAT1, p-STAT3 and p-STAT5 in BMDC following CSE intervention (*p*  < 0.05), suggesting an upregulation of the RAGE/JAK/STAT pathway by CSE (Figure 8). In comparison to the CSE group, the levels of RAGE, p-JAK2, p-STAT1, p-STAT3 and p-STAT5 were significantly decreased in BMDC of the CSE + FPS − ZM1 group (*p*  < 0.05), indicating that the RAGE inhibitor FPS-ZM1 effectively suppressed the CSE-induced activation of the JAK/STAT pathway. In the CSE + AG490 group, the levels of p-JAK2, p-STAT1, p-STAT3 and p-STAT5 decrease compared to the CSE group (*p*  < 0.05), while no difference in RAGE expression was observed between the groups. This suggests that AG490, a JAK2 inhibitor, attenuates BMDC activation following CSE exposure without affecting RAGE expression, while it decreases the expression of p-JAK2, p-STAT1, p-STAT3 and p-STAT5 in BMDC.

### 3.8. Secretion of IL-6 and IL-1β by BMDC

**Figure 9:**
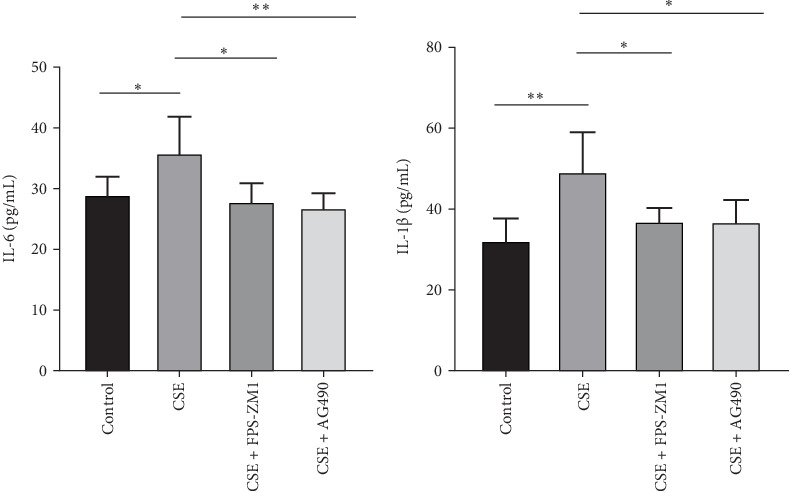
The effects of FPS-ZM1 and AG490 on the secretion of IL-6 and IL-1β in BMDC measured by ELISA. Levels of IL-6 (A) and IL-1β (B) in BMDC in four groups. *⁣*^*∗*^*p* < 0.05, *⁣*^*∗∗*^*p* < 0.01.

ELISA assays demonstrated a significant increase in IL-6 and IL-1β concentrations in the cellular supernatants post-CSE exposure compared to the control group. Both the RAGE inhibitor FPS-ZM1 and the JAK2 inhibitor AG490 significantly reduced the secretion of IL-6 and IL-1β (*p*  < 0.05, Figure 9). These findings suggest that CSE enhances the release of cytokines IL-6 and IL-1β from BMDCs, whereas FPS-ZM1 and AG490 inhibit CSE-induced BMDC activation, leading to decreased IL-6 and IL-1β secretion and a reduced inflammatory response.

## 4. Discussion

Ongoing chronic inflammation is crucial to the development of COPD, with the activation and disruption of adaptive immunity being the primary mechanisms involved. Individuals with COPD demonstrate an increased presence of diverse inflammatory cells in their lungs with DCs playing a pivotal role in bridging the body's innate and adaptive immune responses. A comprehensive understanding of the activation and underlying mechanisms of DCs is essential.

Current evidence indicates that DCs facilitate the proliferation of Th1 and Th2 cells [34] and activate B cells through antigen presentation [19]. Our findings indicate that tobacco exposure leads to an upregulation of CD40 and CD86 expression in mDCs, aligning with our prior research and suggesting that such exposure fosters the maturation and activation of mDCs in the lungs. Furthermore, levels of CD40 and CD86 increased following 24 h of CSE treatment in BMDC cultures, demonstrating that cigarette smoke exposure activates DCs and elicits innate immune responses both in vivo and in vitro. Additionally, chronic exposure to tobacco elevated levels of CD69, CD86 and CD138 on B cells in murine lung tissue, suggesting that long-term tobacco smoke exposure enhanced the activation and differentiation of B cells into plasmablasts in lungs, thereby stimulating adaptive immunity. Despite ongoing research, the dynamic interactions and crosstalk between the activation of mDCs and B cells remain insufficiently understood.

It is widely recognised that prolonged exposure to tobacco causes to the accumulation of inflammatory mediators in lung tissue, which may result in the formation of AGEs and subsequent activation of the ligand-RAGE pathway [35]. We have shown in earlier studies that cigarette smoke exposure induced neutrophil NETosis, depolymerisation of nuclear chromatin structure, and release of large amounts of HMBG1 from the nucleus, which bound to DNA to form HMGB1:DNA complexes [36, 37]. HMGB1 interacts with RAGE and TLR4 on the surface of DCs, promoting their maturation and activation, which subsequently induces T cell differentiation, while RAGE^−/−^ DCs could not be activated by HMGB1 [17]. Additionally, another research indicated that RAGE activation triggers the downstream STAT3 signalling pathway in A549 cells, and that silencing RAGE directly suppresses autophagic apoptosis in alveolar epithelial cells, thereby mitigating lung inflammation [38]. In alignment with these observations, our findings identified an upregulation of RAGE and STAT1 genes in lung tissue, alongside increased expression levels of RAGE, p-JAK2, p-STAT1, p-STAT3 and p-STAT5 in pulmonary mDCs in a smoking-induced mouse model of emphysema.

Subsequent suppression of RAGE in DCs in mice resulted in a reduction of the heightened responses of mDCs and B cells, as well as a decrease in the expression of p-JAK2, p-STAT1, p-STAT3 and p-STAT5 in mDCs, indicating a connection between RAGE signalling and the function of mDCs and B cells. Additionally, in vitro studies demonstrated increased expression levels of RAGE, p-JAK2, p-STAT1, p-STAT3 and p-STAT5 in BMDC following CSE stimulation. Interestingly, our in vitro findings revealed that inhibition of RAGE and JAK2 resulted in diminished BMDC activation, while the increase in RAGE, p-JAK2, p-STAT1, p-STAT3 and p-STAT5 caused by CSE was inhibited by a RAGE inhibitor. However, inhibition of JAK2 did not affect RAGE expression, suggesting that RAGE functions upstream of the JAK/STAT pathway, and mDCs activation is mediated through the up-regulation of the RAGE/JAK/STAT pathway induced by cigarette smoke exposure.

RAGE, acting as an inflammatory mediator, triggers the downstream JAK/STAT pathway to maintain and amplify inflammation in COPD, with IL-6 and IL-1β being key contributors [39–41]. Reduction of RAGE expression in alveolar macrophages, key signalling mediators p38 and NF-κB were suppressed, leading to a decrease secretion of TNF-α and IL-1β [42]. The severity of emphysema is associated with a continuous and prolonged inflammatory reaction. Our research demonstrated that levels of IL-6 and IL-1β increased following cigarette smoke exposure both in vivo and in vitro. The reduction of RGAE in DCs in mice, or the inhibition of RAGE or JAK2 in BMDC in vitro, led to decreased secretion of IL-6 and IL-1β, aligning with earlier findings. Moreover, increased Lm and formation of emphysema were observed in mice following chronic tobacco smoke exposure, while a conditional knockdown of RAGE in DCs led to a reduction in Lm and less emphysema. This suggests a protective effect of RAGE knockdown in DCs against cigarette-associated emphysema in mice.

Cigarette smoking triggers the generation of significant quantities of reactive oxygen species within lung tissue, which in turn initiates the creation and release of DAMPs and inflammatory agents, leading to lung inflammation [43]. Nonetheless, recent research indicates that inflammation in the airways and lungs persists in COPD patients even after they quit smoking, and the destruction of lung tissue continues to advance [44]. Therefore, a critical consideration is the mechanism underlying persistent chronic inflammation in the lungs without smoke stimulation. The infiltration of immune cells after smoke stimulation contributes to alveolar destruction [9], and then innate immunity initiates intrinsic immunity, causing persistent inflammation of the lungs. Our previous study identified a significant infiltration of mature, overactivated DCs in the lungs of COPD patients and cigarette smoke-exposed emphysema mice, which highly expressed costimulatory molecules and HLA-DR and had a strong antigen-presenting function [31]. In this study, we found that hyperactivation of pulmonary mDCs and B cells accompanied by elevated serum inflammatory factors in experimental emphysema murine models, implying initiation of immune responses and persistent inflammation responses.

RAGE is a receptor that binds to multiple ligands generated by tobacco and contributes to the progression of COPD [45]. Studies have demonstrated that cells treated with CSE modify the localisation and levels of RAGE, playing a role in COPD development by triggering redox-sensitive DAMPs through MAPK and NF-κB pathways [46]. In transgenic mice overexpressing RAGE, there is a notable enlargement of alveolar spaces and a rise in cell apoptosis, whereas administering RAGE inhibitors to mice halts emphysema development [47]. Our findings indicate that prolonged exposure to tobacco triggers the development and activation of lung mDCs and B cells by enhancing the RAGE/JAK/STAT pathway in mice, as well as stimulating inflammatory responses in peripheral blood and leading to emphysema formation. The knockdown of RAGE in DCs resulted in the suppression of mDCs and B cells activation, downregulation of the RAGE/JAK/STAT pathway in mDCs, and conferred protection against emphysema in mice subjected to chronic tobacco exposure. Besides, in vitro, inhibition of RAGE and JAK2 in BMDC partially antagonised CSE-induced activation of BMDC by downregulating the RAGE/JAK/STAT pathway and reducing the production of inflammatory factors. Consequently, we propose the hypothesis that targeting the RAGE may serve as an effective therapeutic strategy for emphysema associated with tobacco use.

The limitation of this study is that we only use CD11c and MHC II staining to identify mDCs (CD11c^+^MHC Ⅱ^+^) from single-cell suspensions of lung tissue. Some alveolar macrophages and plasmacytoid DCs may express CD11c and MHC II. Furthermore, interstitial macrophages and DCs in lung tissue share the expression of CD11c and MHC II. This overlapping expression may have influenced the results of our experiments, representing a limitation of our study. In addition, our study primarily focused on the analysis of mDCs and B cells, while quantifying alveolar macrophages, neutrophils, eosinophils, and T cells in lung cells was not feasible. Consequently, our findings do not fully encapsulate the overall inflammatory landscape. In future research, we aim to enhance the comprehensiveness and precision of our experimental approach to gain a more holistic understanding of inflammatory processes.

## 5. Conclusion

To sum up, our study demonstrates that cigarette smoke exposure activates mDCs by upregulating the RAGE/JAK/STAT pathway, which may subsequently induce irregular B cell-mediated immune responses in the lungs of mice with emphysema. Moreover, the activation of mDCs and lung inflammation caused by cigarette smoke are mitigated when RAGE expression is either absent or down-regulated on DCs, both in vitro and in vivo. These results suggest a potential therapeutic approach for COPD and emphysema caused by smoking.

## Data Availability

The data that support the findings of this study are available from the corresponding author upon reasonable request.
